# BET-inhibitor DYB-41 reduces pulmonary inflammation and local and systemic cytokine levels in LPS-induced acute respiratory distress syndrome: an experimental rodent study

**DOI:** 10.1186/s40635-024-00604-z

**Published:** 2024-02-26

**Authors:** Manuela Iten, Camille Gschwend, Alessandro Ostini, David Robert Cameron, Christine Goepfert, David Berger, Matthias Haenggi

**Affiliations:** 1 Department of Intensive Care Medicine, Inselspital, University Hospital Bern, Freiburgstrasse 16, 3010 Bern, Switzerland; 2 Department of Intensive Care Medicine, Cantonal Hospital Aarau, Tellstrasse 25, 5001 Aarau, Switzerland; 3 COMPATH, Institute of Animal Pathology, Vetsuisse Faculty, University of Bern, Laenggassstrasse 122, 3012 Bern, Switzerland

**Keywords:** ARDS, BET-inhibitor, Acute lung injury, Lung fibrosis

## Abstract

**Background:**

Acute respiratory distress syndrome (ARDS) is a form of respiratory failure stemming from various underlying conditions that ultimately lead to inflammation and lung fibrosis. Bromodomain and Extra-Terminal motif (BET) inhibitors are a class of medications that selectively bind to the bromodomains of BET motif proteins, effectively reducing inflammation. However, the use of BET inhibitors in ARDS treatment has not been previously investigated. In our study, we induced ARDS in rats using endotoxin and administered a BET inhibitor. We evaluated the outcomes by examining inflammation markers and lung histopathology.

**Results:**

Nine animals received treatment, while 12 served as controls. In the lung tissue of treated animals, we observed a significant reduction in TNFα levels (549 [149–977] pg/mg vs. 3010 [396–5529] pg/mg; *p* = 0.009) and IL-1β levels (447 [369–580] pg/mg vs. 662 [523–924] pg/mg; *p* = 0.012), although IL-6 and IL-10 levels showed no significant differences. In the blood, treated animals exhibited a reduced TNFα level (25 [25–424] pg/ml vs. 900 [285–1744] pg/ml, *p* = 0.016), but IL-1β levels were significantly higher (1254 [435–2474] pg/ml vs. 384 [213–907] pg/ml, *p* = 0.049). No differences were observed in IL-6 and IL-10 levels. There were no significant variations in lung tissue levels of TGF-β, SP-D, or RAGE. Histopathological analysis revealed substantial damage, with notably less perivascular edema (3 vs 2; *p* = 0.0046) and visually more inflammatory cells. However, two semi-quantitative histopathologic scoring systems did not indicate significant differences.

**Conclusions:**

These preliminary findings suggest a potential beneficial effect of BET inhibitors in the treatment of acute lung injury and ARDS. Further validation and replication of these results with a larger cohort of animals, in diverse models, and using different BET inhibitors are needed to explore their clinical implications.

## Take home message

Early treatment with BET inhibitor Dyb-41 reduced cytokine levels in lung tissue of experimental ARDS. If BET inhibitors may have a potential effect in the treatment of ARDS is worth further investigation.

## Background

Acute respiratory distress syndrome (ARDS) is a serious medical condition marked by lung inflammation and accumulation of fluid, leading to severe breathing difficulties and organ failure [1]. It can emerge as a complication of various diseases, such as pneumonia, sepsis, and trauma, and often leads to high mortality rates. The pathogenesis of ARDS involves the dysregulation of several cellular pathways, including the upregulation of inflammatory cytokines and consequently leading to the disruption of the alveolar–capillary barrier. Structural alterations encompass widespread alveolar impairment in the initial phases and subsequent fibrosis during the later stages. Numerous attempts involving pharmacological treatments have not yielded improvements in survival [1]. Steroids, owing to their potential for reducing inflammation and fibrosis, might offer some limited therapeutic advantages, particularly during the advanced phases of the illness [2].

Epigenetic modulation, achieved through histone acetylation/deacetylation, governs the transcriptional regulation of critical genes associated with inflammation (such as IL-1beta, IL-6, TNF-alpha) or fibrosis (like collagen, actin). Bromodomain extra-terminal (BET) proteins have a firm affinity for acetylated lysine residues on histones, resulting in the liberation of DNA from histone grip and facilitating gene transcription [3]. Recently, inhibitors targeting BET proteins have displayed potential in countering inflammation and fibrosis, as evidenced by their ability to prevent mortality in cases of lipopolysaccharide (LPS)-induced septic shock [4–6]. This therapeutic impact has been linked to the suppression and activation of inflammatory gene transcription [4, 5, 7]. For instance, treatment of macrophage cells with I-BET impeded the activation of a specific subset of LPS-induced genes, including those encoding cytokines, chemokines, and various transcription factors integral to the inflammatory response [5].

The small molecule BET-inhibitor Dyb-41 (Dybli AG, Basel, now Worg Pharmaceuticals Shanghai Europe, Basel, Switzerland) inhibits BET bromodomain proteins (BRD)2, BRD3 and BRD4, but no other bromodomain proteins; with a predominance against BRD4. It exhibits low in vitro toxicity, sufficient oral bioavailability and a short half-life [8]. Preclinical data of the compound and its counterparts like JQ1 and I-BET show various anti-inflammatory effects: the compound suppresses TNFα and IL-6 production cell cultures challenged with LPS, and reduces in a concentration-dependent manner IL-1β, IL-6 and TNFα in another LPS challenged model [9]. BET-inhibitor Dyb-41 compound suppress TNF-α/interferon induced CCL2 expression in astrocytes, and suppress VCAM expression induced by TNFα in brain endothelium (data from Dybi AG, now Worg Pharmaceuticals Shanghai Europe, Basel, Switzerland). These properties make them of particular interest for the possible treatment of the acute respiratory distress syndrome. The aim of our proof-of-concept study is to evaluate the efficacy of the BET-I DYB-41 for early treatment of LPS-induced acute lung injury in a rat model of ARDS.

## Methods

This proof-of-concept study took place at the experimental surgical facility located within the University of Bern, Switzerland. Twenty-four CD® (Sprague Dawley) IGS Rats (Charles River Germany) of both sexes, body weight 250 to 300 g, housed in specific pathogen-free rooms (12 h light/dark conditions, 23 °C ± 1°, water and nutrition ad libitum) were used for this study. Animal protocols were run in accordance with the guidelines of the Swiss Animal Protection Law and were approved by the Cantonal Committee on Animal Experiments of the State of Bern (approval BE 102/2020). This report follows the applicable ARRIVE guidelines.

**Fig. 1:**
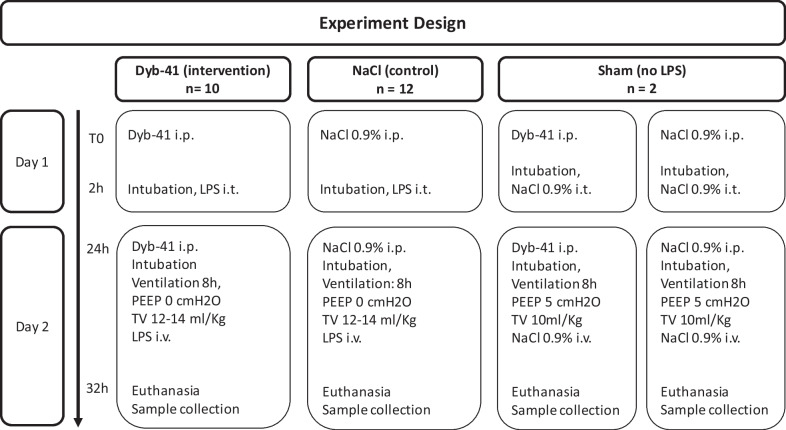
Experiment design. Workflow of the experiment. *n*, number of animals; *h*, hours; *i.p.*, intraperitoneal; *i.t.*, intratracheal; *i.v.*, intravenous; *PEEP* positive end expiratory pressure; *TV* tidal volume

ARDS was induced in 22 rats with a double hit LPS model [10–12] and additional harmful ventilation [13]. Ten rats were assigned to the active Dyb-41 compound and 12 to placebo. Two rats served as controls without ARDS induction (no LPS) for the establishment of reference cytokine levels (Fig. 1). The experiment had to be halted for a duration of 10 months due to ICU staff shortage and to University regulations that prohibited active research amidst the COVID-19 pandemic. This interruption caused supply issues of LPS and rendered proper randomization impossible.

On day 1, 2 h after intraperitoneal (i.p.) administration of Dyb-41 (50 mg/kg, diluted in 1% hydroxypropyl-betacyclodextrin to 10 mg/ml) or placebo (normal saline NaCl 0.9% 5 ml/kg) rats were sedated with midazolam 5 mg/kg and fentanyl 20 mcg/kg i.p., subsequently placed in an induction chamber and anesthetized with sevoflurane. After intubation (G14 angiocath-BD Venflon, BD Switzerland), correct placement of the endotracheal tube was confirmed using capnography. LPS [*E. coli* O55:B5 (Merck/Sigma-Aldrich, Darmstadt, Germany); 500mcg/kg, diluted in NaCl 0.9% to 150 µl] was administered intratracheally (i.t.). Anesthesia was then interrupted and the animals were extubated and placed back into their cages (Fig. 1). On day two, a second i.p. dose of Dyb-41 50 mg/kg or placebo was administered and rats were sedated, anaesthetized and intubated following the same protocol as on day one. Anesthesia was maintained by sevoflurane and addition of remifentanil after establishing a vascular access by inserting a pediatric double lumen central venous catheter (Arrow Blue FlexTip, 4 Fr × 5 cm, Teleflex Medical, Westmeath, Ireland) into the right jugular vein. Through the second lumen LPS was infused at a dose of 5 mg/kg over an hour. Animals were ventilated for a total of 8 h in a volume controlled mode with a tidal volume of 12–14 ml/kg, 0 cmH_2_O of PEEP, a respiratory rate of 55–60/min on 100% F_I_O_2_ and inspiration/expiration ratio of 1:2 [13] with ventilators for small animals (Ventelite, Harvard Apparatus, Holliston, Massachusetts, USA) (Fig. 1).

One sham animal received intraperitoneal Dyb-41 Compound, the other NaCl 0.9% on day one, prior to intubation and i.t. inoculation with NaCl 0.9% as placebo. On day two, Dyb41 compound or NaCl 0.9% were administered i.p., followed by intubation and i.v. application of placebo (NaCl 0.9%). Sham rats were ventilated more protectively using smaller tidal volumes (10 ml/kg) and a PEEP of 5 cmH2O, respiratory rate and FiO2 was set the same as ARDS rats (Fig. 1).

After 8 h of mechanical ventilation all animals were euthanized with pentobarbital before blood and tissue sampling. Blood samples were collected in EDTA tubes, spun at 1000 g, and then stored at − 80 °C until further analysis. Lungs were collected and separated into individual lobes. For histology, two lobes were fixed in formalin (4% in phosphate buffered saline) for 24 h and then embedded in paraffin. Two to three μm slices were cut and stained with hematoxylin–eosin. The other lobes were immediately frozen in liquid nitrogen and stored at − 80 °C until further use.

A fully trained veterinary pathologist assessed histological lung slices in a blinded fashion using two well-established semi-quantitative histopathologic scoring system for lung injury described by Zeldin et al. [14] and Matute-Bello et al. [15].

Levels in blood and homogenized lung tissue for IL1-β, IL-6, and IL-10 were measured with rat-specific ELISA kits for lysates and plasma (Invitrogen, Waltham, Massachusetts) according to manufacturer’s instructions. TGF-β (MyBioSource, San Diego, California), SP-D (Biomatik, Ontario, Canada) and RAGE (Abcam, Cambridge UK) were also measured with specific ELISA kits. Results from homogenized lung tissue were calculated in relation to the measured total protein levels in the sample. Total protein was extracted with use T-Per tissue protein extraction reagent + protease inhibitor and quantified with the Pierce BCA protein assay kit (ThermoFisher, Waltham, Massachusetts, USA).

### Statistics

Data were analyzed with GraphPad Prism (version 8.0.1 for Windows, GraphPad Software, San Diego, CA, USA). Data are reported as median with interquartile range. Differences between groups were determined using the Mann–Whitney test. A two-tailed alpha level of < 0.05 was considered significant. The exploratory nature of this project made an a priori sample size calculation impossible.

## Results

**Fig. 2:**
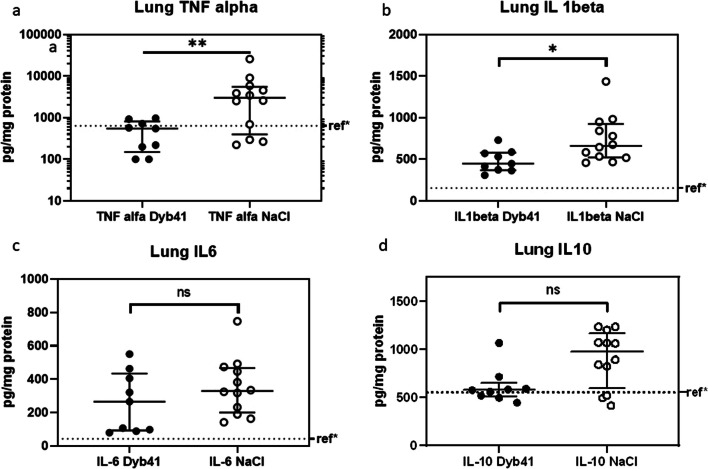
**a**–**d** Cytokine levels from homogenized lung tissue. The reference level (ref*) was derived from homogenized tissue samples from two control animals not exposed to LPS

Twelve animals treated with placebo and nine animals with the active compound entered the analysis. One set of samples in the Dyb41 group was lost. Significant reductions in TNF-α for the treatment group (549 [149–977] pg/mg vs. 3010 [396–5529] pg/mg; *p* = 0.009) and IL-1β (447 [369–580] pg/mg vs. 662 [523–924] pg/mg; *p* = 0.012) were observed in the homogenized lung tissue compared to the placebo group. In the Dyb-41 group we saw a trend for lower IL-10 (576 [505–653] pg/mg vs. 973 [593–1166] pg/mg; *p* = 0.07) compared to the placebo group. No difference was found for IL-6 (266 [93–434] pg/mg vs. 330 [200–467] pg/mg; *p* = 0.25) (Fig. 2). Lung tissue levels for TGF-β (0.06 [0.043–0.078] ng/g vs. 0.056 [0.052–0.0687] ng/g; *p* = 0.73), SP-D (66.9 [46.9–86.9] ng/g vs. 57.5 [37.0–65.5] ng/g; p = 0.28) and RAGE (1.61 [1.16–3.26] µg/g vs. 2.18 [1.45–3.74] µg/g; *p* = 0.41) showed no differences between intervention and control groups.

**Fig. 3:**
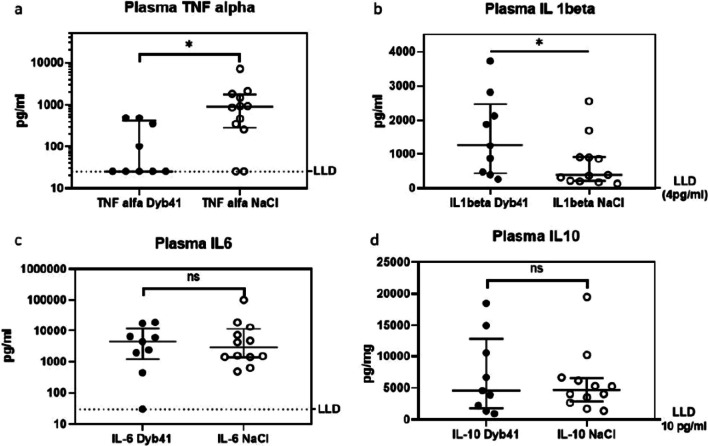
**a**–**d** Cytokine levels in plasma. LLD denotes lower level of detection

TNF-α in the plasma was significantly reduced in ARDS rats treated with Dyb41 compared to those receiving placebo (25 [25–424] pg/ml vs. 900 [285–1744] pg/ml, *p* = 0.016). IL-1β in plasma was significantly higher in the treated animals (1254 [435–2474] pg/ml vs. 384 [213–907] pg/ml, *p* = 0.049) than in control animals. No difference was found in plasma levels for IL-6 (4452 [1200–12307] pg/ml vs 2912 [1406–11845] pg/ml, *p* = 0.92) and IL-10 (4569 [1784–12780] pg/ml vs 4649 [2869–6538] pg/ml, *p* = 0.97 (Fig. 3). The same hold true for plasmatic TGF-β (58 [37–66] pg/mL vs. 67 [46–87] pg/mL; *p* = 0.23, SP-D (992 [440–1657] ng/mL vs. 1978 [262–2165] ng/mL; *p* = 0.23) and RAGE (8 [4.9–20.5] pg/mL vs. 35 [5.7–1508] pg/mL; *p* = 0.11).

**Fig. 4:**
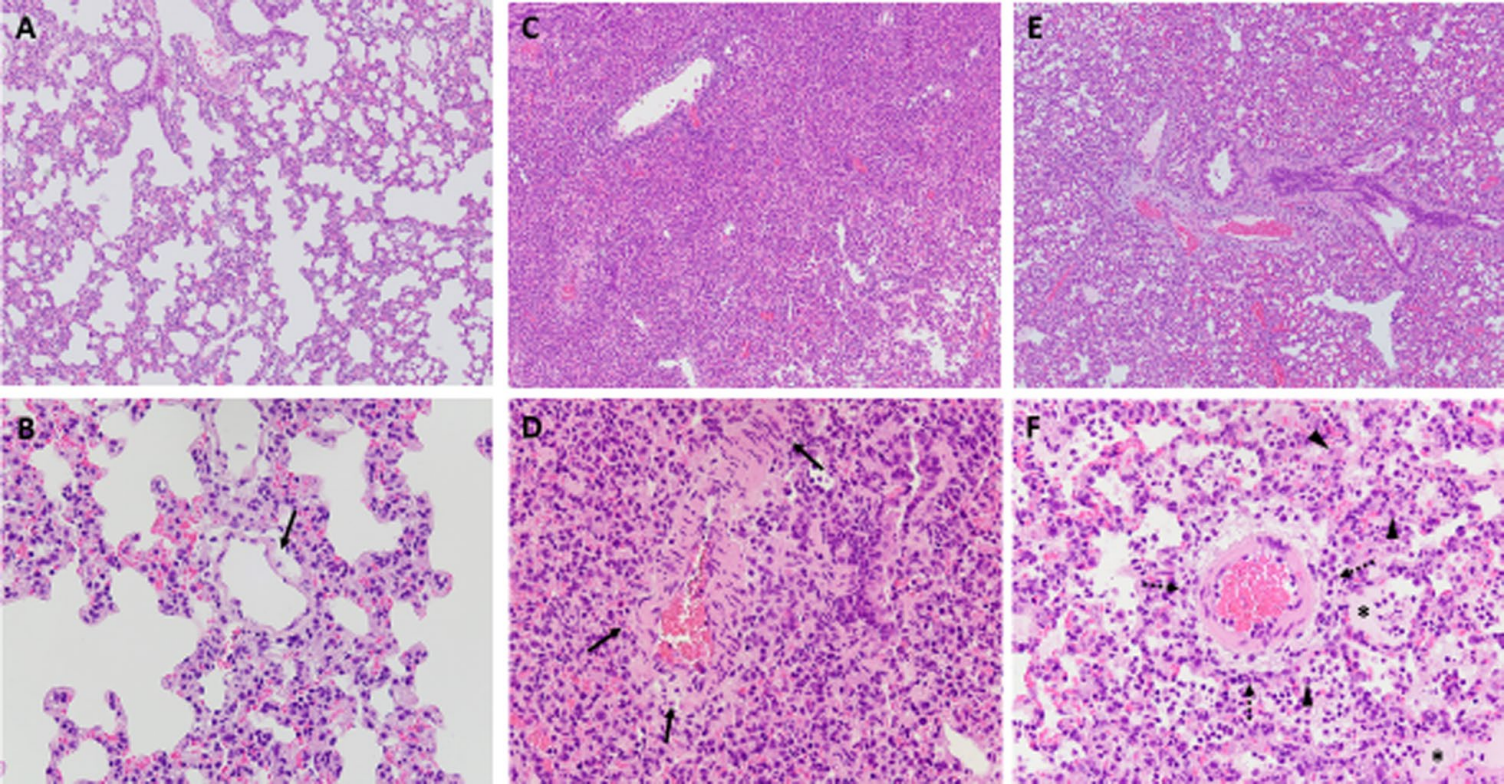
**a**–**f** Representative histopathologic images; HE stain, 100× (**A**, **C**, **E**) and 400× (**B**, **D**, **F**) magnification. **A**, **B** Reference animal, no LPS, placebo (ventilation only): mild perivascular edema (arrow). **C**, **D** Control animal; the widened perivascular space is occupied by numerous neutrophils (arrow) and macrophages, which are also present in surrounding alveoli. **E**, **F** Animal with DYB-41: infiltration of neutrophils and early cellular necrosis (acute, arrow), alveolar macrophages (arrowhead) and moderate alveolar edema (asterisk)

Animals induced with LPS exhibited elevated histopathology scores compared to the two sham animals (8, [6–9] vs 3.5, [3, 4]) [14]. LPS induction resulted in acute to subacute and moderate to severe bronchoalveolar pneumonia, characterized by macrophages and neutrophils completely occupying the interstitium and alveolar space (Fig. 4c, d).

Some animals treated with Dyb-41 displayed a more acute inflammatory reaction pattern with predominantly neutrophils, which, in tendency, were less numerous, thereby leaving more alveolar space compared to the control group (Fig. 4e, f). In contrast, very few inflammatory cells were visible in the lung of Sham animals (Fig. 4a, b).

Dyb-41 significantly reduced perivascular edema (3 [2, 3] to 2 [2], *p* < 0.05). The total score decreased from 8.5 [8, 9] to. 8 [7, 8], but did not reach statistical significance, *p* = 0.065 [14]. Semi-quantitative scoring of the pulmonary changes using a distinct scoring schemes did not reveal significant differences in the total score between the Dyb-41 and control group (Dyb-41 7210 [6845–8365] vs Control 7043 [6756–7664] *p* = 0.032) [15].

## Discussion

As a main result, this proof-of-concept study successfully validated our hypothesis that the pharmacological inhibition of BET-proteins reduces inflammatory cytokine levels in a rodent model of LPS-induced acute respiratory distress syndrome. However, it’s important to note the inconsistent behavior of these cytokines.

While TNFα levels in both lung tissue and serum of treated animals were lower compared to the untreated group, the findings for IL-1β were mixed. Lower levels were noted in the lung tissue of the Dyb-41 group, while higher values were observed in the blood samples compared to the placebo group. Suppressive effects of DYB-41 on IL-1β, TNFα and IL6 were documented for human blood exposed to LPS (unpublished data by Dybli AG, now Worg Pharmaceuticals, LPS challenge done by Centre for Human Drug Research (CHDR); Leiden, the Netherlands, https://chdr.nl/). Consequently, our findings regarding TNF-α align with the in vitro study of DYB-41, and the congruence extends to IL-1β levels in lung tissue. However, the observed elevation of IL-1β in the blood of treated animals remains unclear. One plausible explanation is that BET inhibitors, such as DYB-41, may influence the expression of both inflammatory and anti-inflammatory cytokines, resulting in a net effect that is not yet fully comprehended. Furthermore, the timing of cytokine measurements in ARDS plays an important role [16] and the histopathology of the lungs revealed a broader variation in the extent of lung injury and stage of pneumonia compared to the control animals. The small number of animals in the study might also contribute to inconsistent results.

Treatment with Dyb-41 led to a notably reduced presence of perivascular edema compared to the placebo group. Despite a lower overall histopathology score in treated animals, this difference did not reach statistical significance]. One plausible explanation is that this scoring system was initially designed for obstructive lung disease, lacking specificity for ARDS. Notably, even the acute lung injury score from the American Thoracic Society, specifically crafted for ARDS assessment in animals, failed to reveal a difference in the overall score. Although neutrophil infiltration appeared visually more pronounced in control animals, neither an area measure [14] nor an absolute count [15] of acute inflammatory cells could accurately capture the obvious difference. In severe acute inflammatory states, such as observed in our project, the scoring system proposed by Matute-Bello proves inadequate, as it scores > 5 neutrophils per field as maximum value [15]. The same limitation applies to the area graduation method outlined by Zeldin et al. [14].

Histopathology scores for assessing ARDS in rodents are not well established and lack wide validation, but several studies confirm a correlation between histology and measured cytokine levels for inflammatory lung injury [17–19]. Our findings support this correlation, demonstrating lower perivascular edema, fewer neutrophils in alveolar space and lower TNF-α and IL-1β levels in lung tissue of Dyb-41 treated animals.

BET-Inhibitors have proven effective in reducing inflammation in rheumatoid arthritis [20], kidney disease [21] and lung fibrosis, either bleomycin or radiation-induced [22, 23]. Additionally, another compound has demonstrated anti-inflammatory effects in a psoriasis model [24] and the bromodomain inhibitor iBET151 has been successful in preventing experimental allergic lung inflammation [25].

While most research on BET inhibitors has focused on chronic inflammation or their activity against cancer, their role in acute settings is less explored [26]. Preemptive administration of I-BET has been shown to protect mice against otherwise lethal effects of experimental LPS challenge and polymicrobial sepsis following cecal ligation and puncture [5]. Furthermore, JQ1 has been found to reduce levels of IL-6 and TNF and prevent death in mice induced with LPS-induced endotoxemia [4].

Our experiments demonstrate that inhibition of Bromodomain-containing protein 4 attenuates pulmonary inflammation caused by LPS suggesting further exploration as a treatment option for ARDS. However, our study has several limitations. The most notable is the lack of randomization. Our study had to be interrupted and paused for several months during the COVID-19 crisis due to university regulations. Additionally, supply issues for LPS were encountered. Nevertheless, outcome assessment of histology and cytokine analyses was blinded. The double hit LPS-model only represents one specific model of the ARDS syndromatic complex and whether BET-I would show similar effects in differing ARDS models remains to be determined. We did not examine the pharmacokinetic or pharmacodynamic profile of DYB-41 and the dosing regimen relied on initial data provided by Dybli Pharma. It is uncertain whether a higher dosage or an alternative dosing schedule might have produced a more robust response. Furthermore, it is crucial to note that our rat model replicates the initial stages of ARDS development, so whether the trajectory of ARDS is genuinely influenced by the compound in its early stages, or if it has any impact on the subsequent fibrosis phase, remains a matter of speculation. Of note, the simultaneous administration of the compound and LPS does not represent a valid clinical scenario.

## Conclusion

In conclusion, our pilot study successfully revealed a moderate impact of Dyb-41, a BRD4 inhibitor, in a rat model of LPS-induced ARDS. These findings suggest that BET inhibitors hold promise as a potential therapeutic approach for treating ARDS. However, further research is essential to thoroughly evaluate their safety and efficacy before advancing to clinical trials.

## Data Availability

The datasets used and/or analyzed during the current study are available from the corresponding author on reasonable request.

## Abbreviations

ARDS: Acute respiratory distress syndrome
BET: Bromodomain and extra-terminal domain proteins
BET-I: Bromodomain and extra-terminal domain protein inhibitor
BRD: BET bromodomain proteins
cm: Centimeter
DNA: Desoxyribonucleic acid
ELISA: Enzyme-linked immunosorbent assay
HSSLI: Histopathologic scoring system for lung injury
IL: Interleukin
i.p.: Intraperitoneal
i.t.: Intratracheal
i.v.: Intravenous
kg: Kilogram
LPS: Lipopolysaccharides
mg: Milligram
min: Minute(s)
ml: Milliliter
pg: Pictogram
RAGE: Receptor for Advanced Glycation End-products
SP-D: Surfactant protein D
TGF-β: Transforming growth factor β
TNF: Tumor necrosis factor
